# Intralymphatic histiocytosis of the upper eyelid in a patient of Korean descent: A case report

**DOI:** 10.1097/MD.0000000000036035

**Published:** 2023-11-10

**Authors:** Dong Hee Ha, Hee Sung Kim, Jeong Kyu Lee

**Affiliations:** a Department of Ophthalmology, College of Medicine, Chung-Ang University Hospital, Seoul, South Korea; b Department of Pathology, College of Medicine, Chung-Ang University Hospital, Seoul, South Korea.

**Keywords:** eyelid edema, intralymphatic histiocytosis, triamcinolone

## Abstract

**Rationale:**

Diagnosing intralymphatic histiocytosis can be challenging due to its rarity. We present a case of intralymphatic histiocytosis in the upper eyelid of a Korean patient. We treated the condition by surgical debulking and intralesional triamcinolone injection.

**Patient concerns:**

A 59-year-old man was referred to our clinic with a 7-year history of unilateral swelling in the right upper eyelid. He had previously been treated with long-term oral steroids and immunosuppressants, but his eyelid swelling persisted. Unilaterally non-pitting erythematous edema was localized on the right upper eyelid without any itching or pain. His best corrected visual acuity at presentation was 20/20 for both eyes. Enhanced orbital computerized tomography revealed edematous soft tissue thickening in the right upper eyelid. In the laboratory testing, the erythrocyte sedimentation rate showed an increase of 19, and the antinuclear antibody titer was positive with a homogeneous pattern.

**Diagnoses:**

We diagnosed the patient with intralymphatic histiocytosis based on the histopathological findings.

**Intervention:**

We attempted surgical debulking and biopsy on the right upper eyelid due to the persistent symptoms and the absence of a definitive diagnosis.

**Outcomes:**

The patient has demonstrated significant improvement after receiving an intralesional triamcinolone injection in the right upper eyelid following the surgery and is currently under follow-up with no signs of recurrence.

**Lesson:**

Ophthalmologists should consider intralymphatic histiocytosis in cases of persistent eyelid swelling that do not respond to treatment, even in Asian patients. Surgical debulking and intralesional triamcinolone injections may be beneficial for improvement.

## 1. Introduction

Intralymphatic histiocytosis is a rare skin condition characterized histopathologically by prominently dilated lymphatic vessels. It can clinically present with nonspecific cutaneous symptoms. It is difficult to diagnose a condition in which eyelid edema is the only clinical manifestation because it is very rare for periocular swelling to be the sole symptom.^[1]^ The pathogenesis of intralymphatic histiocytosis appears to be associated with chronic inflammation of the affected area, although the exact cause is undetermined. Intralymphatic histiocytosis results in lymph stasis and the subsequent development of lymphangiectasis. It has sometimes been discovered as an incidental histopathological finding. Here, we report a case of intralymphatic histiocytosis presenting as isolated unilateral eyelid edema in a patient of Korean descent.

## 2. Case presentation

**Figure 1.**
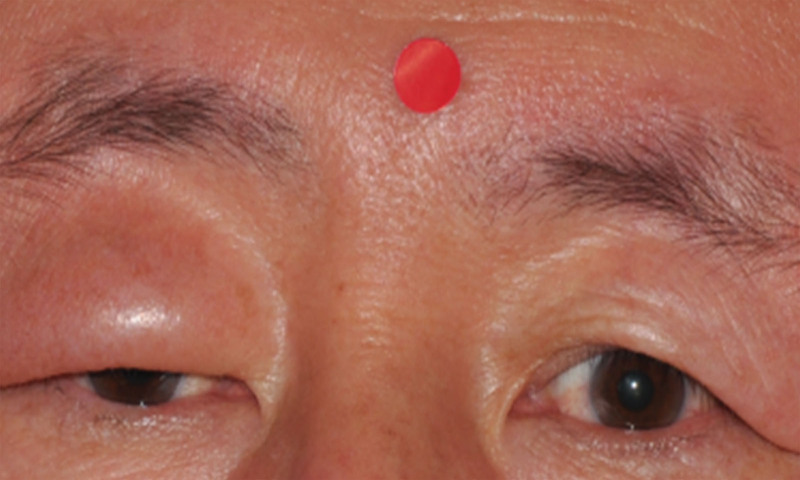
A photograph showing non-pitting and erythematous swelling of the right upper eyelid.

A 59-year-old man was referred to our clinic with a 7-year history of unilateral swelling in his right upper eyelid. The patient, of Korean descent, had a history of well-controlled hypertension. He had previously received oral steroid and immunosuppressant treatments at other clinics (although he was unable to confirm the dosages), but the swelling of his eyelids did not improve. Physical examination revealed unilateral erythematous swelling on the right upper eyelid without any itching or pain (Fig. 1). On the initial ophthalmologic examination, the visual acuity was 20/20, and the intraocular pressure was 14 mm Hg in both eyes. Slit-lamp examination revealed corneal opacity in the left eye and cataracts in both eyes. The patient underwent a thorough clinical investigation, including enhanced orbital computed tomography (CT) and comprehensive laboratory tests. All laboratory findings were normal, except for an increase in the erythrocyte sedimentation rate by 19 and a positive antinuclear antibody titer. An orbital CT scan showed edematous soft tissue thickening in the right upper eyelid. The patient did not show any signs of cranial nerve palsy or a fissured tongue. He denied any recent history of infection, allergies, or trauma.

**Figure 2.**
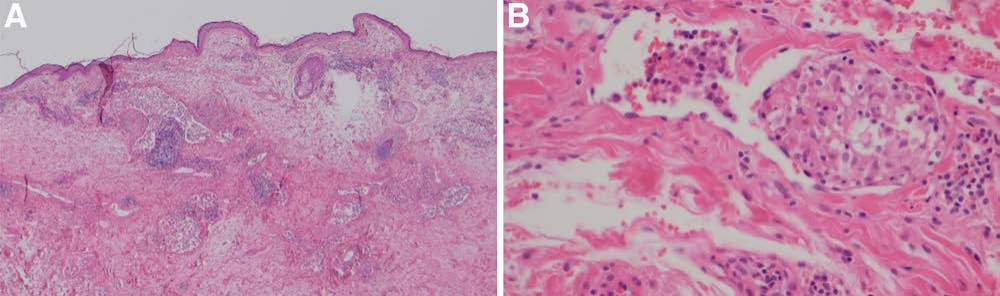
The histopathologic examination of the patient revealed numerous irregularly dilated vessels throughout the dermis (H&E, ×40) (A). The dilated 4 d lymphatic lumen was filled with a chronic granulomatous inflammatory infiltrate of mononuclear epithelioid cells. Dilated vessels also exhibited intraluminal accumulations of mononuclear cells (H&E, ×400) (B). H&E = Hematoxylin and eosin.

**Figure 3.**
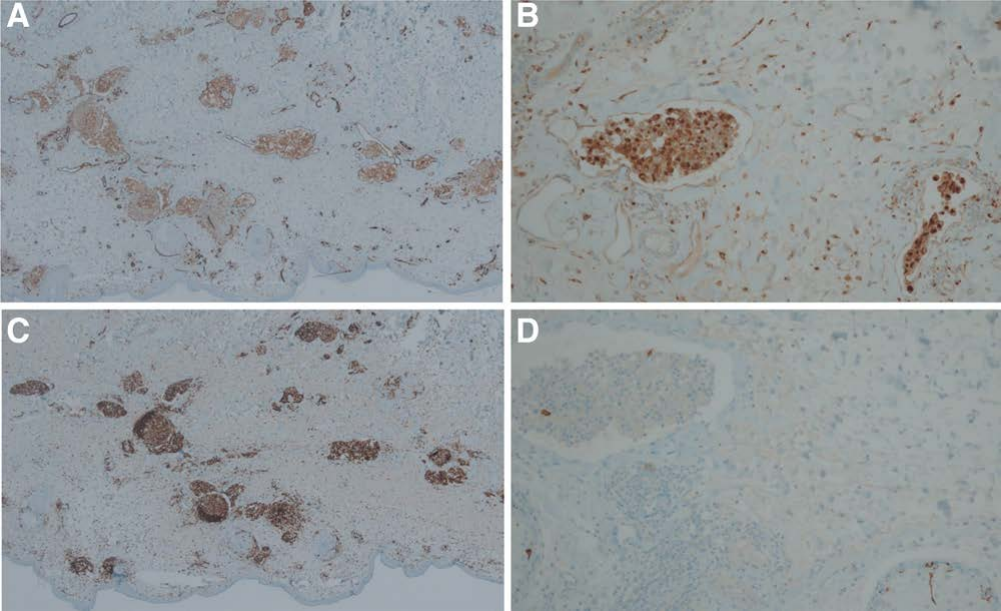
Dilated lymphatics filled with a chronic, non-caseating granulomatous inflammatory infiltrate. The inset demonstrates a CD31-positive endothelium surrounding the infiltrate and confirms its intraluminal location (A). Pathology insets of CD68 (B), CD45 (C), and CD1a (D), respectively.

Due to the persistent edema without a definitive diagnosis, an oculoplastic surgeon attempted surgical debulking and biopsy of the right upper eyelid. Hematoxylin and eosin staining of the specimen revealed a normal epidermis and papillary dermis but prominently dilated vascular structures in the reticular dermis. The dilated lymphatic lumen was filled with a chronic granulomatous inflammatory infiltrate of mononuclear epithelioid cells (Fig. 2A and B). The immunohistochemical investigations demonstrated ectasia of the dermal blood vessels and lymphatics with CD31-positive endothelial cells (Fig. 3A). We observed intravascular and extravascular clusters of CD68-positive/CD45-negative histiocytic-epithelioid cells (Fig. 3B and C). CD1a was positive (Fig. 3D).

**Figure 4.**
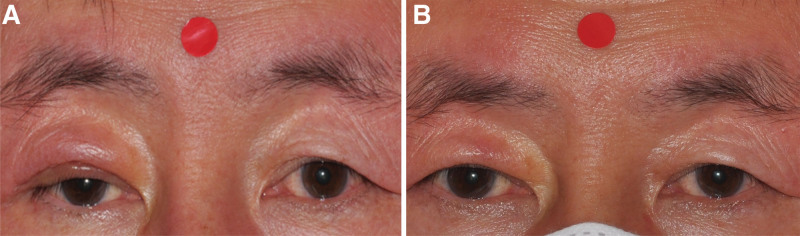
Photographs showed the clinical appearance of a non-pitting and erythematous swelling on the right upper eyelid after surgical debulking (A), followed by intralesional triamcinolone injection (B).

The patient was diagnosed with intralymphatic histiocytosis, and the symptoms partially improved after surgery (Fig. 4A). The patient has shown significant improvement after receiving an intralesional triamcinolone injection in the right upper eyelid following surgery (Fig. 4B). Currently, the patient is under follow-up with no signs of recurrence.

## 3. Discussion

Information regarding intralymphatic histiocytosis is lacking in the ophthalmologic literature. Intralymphatic histiocytosis, first described by O’Grady et al,^[2]^ is an uncommon reactive condition clinically characterized by nonspecific cutaneous manifestations and histopathologically characterized by mononuclear histiocytes accumulation within dilated lymphatic vessels.^[3]^ Numerous cases of intralymphatic histiocytosis have been reported in association with various inflammatory and neoplastic diseases, including rheumatoid arthritis, reactions to metal joint implants, Merkel cell carcinoma, breast cancer, and colon cancer.^[4,5]^ Allergies, chronic inflammation, and lymphangiectasis may be potential causes. Lymphatic drainage obstruction is typically caused by congenital abnormal vessels or damage acquired to the lymphatic vessels from infection, trauma, surgery, or radiation.^[6]^ Several studies have reported that intralymphatic histiocytosis of the eyelid can occur with systemic diseases such as Melkersson-Rosenthal syndrome (MRS) or Morbihan disease.

MRS and Morbihan disease are in the spectrum of diseases associated with persistent facial lymphedema. These conditions, which are both accompanied by intralymphatic histiocytosis, share similar histopathological patterns. Despite the histopathological similarities of non-caseating granulomas, MRS and Morbihan disease often present with different clinical symptoms. While MRS often manifests as lower facial lymphedema involving the lips and tongue,^[7]^ Morbihan disease often demonstrates as upper and bilateral facial lymphedema involving the eyelids with a history of rosacea.^[8]^ Only 8% to 25% of patients with MRS have a complete triad of orofacial edema, facial nerve palsy, and tongue furrowing.^[9]^ Morbihan disease, also known as rosaceous lymphedema or solid persistent facial edema, is an end-stage presentation of rosacea.^[10]^ The research on MRS and Morbihan disease includes reported cases in which patients manifest isolated unilateral eyelid swelling as a possible variant of these 2 diseases.

Similarly, the patient in this study experienced unilateral periocular swelling for 7 years. Without any other histopathologic findings or additional triad features, we concluded that this was a type of intralymphatic histiocytosis. We consulted the dermatology department, which confirmed no evidence of rosacea. This case is similar to previous reports of monosymptomatic periocular MRS. However, all cases reported in Korea were accompanied by orofacial edema, facial nerve paralysis, or other symptoms and were managed without surgical intervention. To the best of our knowledge, this is the first reported case of intralymphatic histiocytosis with unilateral swelling in a patient of East Asian ethnicity with no history of any other inflammatory diseases. The patient underwent surgical debulking for biopsy and cosmetic improvement, followed by an intralesional triamcinolone injection.

The histopathologic examination revealed perilymphatic, intralymphatic, and non-necrotizing granulomatous inflammation. The pathology inset of CD68 revealed that the intraluminal cells were diffuse histiocytes. The presence of CD31-positive endothelium surrounding the infiltrate confirmed their location within the lumen. Lymph stasis may cause inadequate clearance of antigens and localized immune dysfunction, which chronically stimulates histiocyte proliferation and aggregation. This can lead to chronic inflammation. We considered Langerhans cell histiocytosis or Rosai-Dorfman disease as differential diagnoses but ruled them out based on morphological and immunohistochemical findings. Immunohistochemically, the endothelial cells lining the dilated lumina exhibited CD31 immunoreactivity. The intraluminal histiocytes we observed did not exhibit emperipolesis or S-100 protein immunoreactivity. Additionally, CD1a showed reactivity.

Diseases included in the spectrum of persistent facial lymphedema can be difficult to treat. Topical, intralesional, and systemic corticosteroids have been the mainstay of treatment, with varying results.^[11]^ When the disease is associated with an underlying inflammatory condition or malignancy, the intralymphatic histiocytosis may resolve by treating the underlying disorder.^[12]^ Possible treatments include electron beam therapy, cyclophosphamide, topical and systemic steroids, amoxicillin and acetylsalicylic acid, pentoxifylline, NSAIDs, excision, pressure bandages, topical tacrolimus, and methotrexate.^[13]^ We observed partial symptom improvements after surgical debulking and a significant improvement in erythematous edema of the upper eyelid in this patient after intralesional triamcinolone injection. The unilateral eyelid edema responded to intralesional steroid injection. This case suggests that prioritizing surgical debulking followed by intralesional steroid injection can be an effective treatment for isolated eyelid edema, sparing other systemic treatments for recurrence.

## 4. Conclusions

Ophthalmologists encountering patients who suffer from persistent isolated eyelid swelling that does not respond to treatment should consider persistent lymphedema of the face. They should conduct a comprehensive history and physical examination to gather information about the patient’s history of rosacea, the location of the edema, and any other systemic diseases. Active communication with the dermatology and pathology departments can help facilitate a diagnosis. Although there are no specific guidelines for intralymphatic histiocytosis treatment, surgical debulking and intralesional steroid injection can be attempted.

## Acknowledgements

The authors would like to thank the patient for providing informed consent for publication.

## Author contributions

**Conceptualization:** Dong Hee Ha, Jeong Kyu Lee.

**Data curation:** Dong Hee Ha, Jeong Kyu Lee.

**Investigation:** Hee Sung Kim, Jeong Kyu Lee.

**Writing – original draft:** Dong Hee Ha.

**Writing – review & editing:** Hee Sung Kim, Jeong Kyu Lee.
